# Notes and News

**Published:** 1888-03-31

**Authors:** 


### NOTES AND NEWS.

COLLECTED AND COMMUNICATED.

The late Lord Hindlip has bequeathed £1,000, free of legacy duty, to the Burton-on-Trent Infirmary.

---

The application of the Samaritan Hospital for Women, Nottingham, to receive a share of the Hospital Saturday Fund of that town has been refused.

---

It is suggested that a Hospital Sunday Fund should be established in India, to be contributed to and expended for the benefit of the sick in that country, irrespective of creed or caste.

---

It is proposed to endow a bed in the Hastings, St. Leonards and East Sussex Hospital in memory of the late Dr. Adey for twenty-one years a member of the medical staff there, if the necessary sum—£1,000—can be raised.

Mr. Lawson Tait, in a letter to the *Birmingham Post*, expresses the opinion that "hospitals and charities are rapidly ceasing to be charities, and slowly but surely becoming provident institutions in the best sense of the word.

---

The death-rate at the Birmingham and Midland Hospital for Women last year was 5 per cent. Small as this is, it is larger than that of 1885 and 1886, which were respectively 3·7 and 2·8.

---

The workmen of Sunderland have proposed to their employers that they should work a special day's overtime each year, the first wages thus earned to be devoted to the building fund of the Infirmary, and all successive moneys to go to the maintenance of it.

---

Owing to the munificence of the late John and Rebekah Foord, the wards in the west wing of St. Bartholomew's Hospital, Rochester, have been painted, renovated, and refurnished, without any demand being made on the funds of the hospital.

---

The thirtieth annual meeting of the Dental Hospital of London was held at the hospital, Leicester Square, last week, under the presidency of Thomas Arnold Rogers, Esq., one of the vice-presidents. In the report, which was unanimously adopted, the Managing Committee congratulated the governors on the continued success and prosperity of the institution; also on the great benefits which the hospital continues to afford to the suffering poor, 47,441 cases having been treated during the year 1887, a large number of them painlessly (under anæsthetics), being 3,696 in excess of those of the previous year, and 15,537 in excess of the number treated in 1874, when the hospital was removed to its present site. It was stated that it had been found absolutely necessary, in consequence of the continued increase in the number of patients, to further enlarge the hospital by the purchase of the adjoining house for £5,500, which necessitated increasing the mortgage debt on the hospital to £5,500. That a special appeal had been issued for the funds necessary for fitting up and adapting the new wing, and for redeeming the mortgage debt of £5,500 on the hospital building, and the Managing Committee gratefully acknowledged £1,949 3*s.* 6*d.* in response, including £1,000 from the medical staff and lecturers, and £500 from Messrs. Claudius Ash and Sons. That there is still a deficit of £5,700 in the extension account, and the committee are compelled to make a further special appeal for the funds necessary to pay off this debt, which presses so heavily upon the charity, and materially curtails benefits it would otherwise be enabled to confer upon the suffering poor. Help by an increase of annual subscriptions is also earnestly solicited. The charity is endowed, and increased funds would enable it to greatly extend its usefulness.

THE following vacancies are announced this week, the amount of salary in each case being given after the name of the institution :—

**Resident Medical Officers.**

Bethnall House Asylum, E. Junior Medical Officer.—£100, with board, etc.
Borough Asylum, Birmingham. Clinical Assistant.—Board and residence.
Bristol City Lunatic Asylum. Second Medical Officer.—£150, with furnished rooms, board, and washing.
British Seaman's Hospital, Cronstadt, St. Petersburg. Resident Medical Officer.—£180, with furnished apartments.
Derby Borough Asylum. Medical Superintendent.—£350, with furnished rooms, etc.
Essex Lunatic Asylum, Brentwood. Temporary Assistant Medical Officer, for three months.—£30 for the term, with board and washing.
General Infirmary, Northampton. House Surgeon.—£125, with board, etc.
Hospital for Consumption and Diseases of the Chest, Mount Pleasant, Liverpool. Medical Officer.—£70.
Infirmary for Children, Myrtle Street, Liverpool. House Surgeon.—£85, with board.
Liverpool Dispensaries. Two Assistant Surgeons.—£80, with board, lodging, etc.
Royal Union Ophthalmic Hospital, Moorfields, E.C. Junior House Surgeon.—£50.

**Non-resident Medical Officers.**

Westminster Hospital. Medical Registrar.—£40.
West London Hospital, Hammersmith Road. Clinical Assistants.
St. Peter's Hospital for Stone, Henrietta Street, W.C. Anæsthetist.—£50.

**Matron.**

Paddington Green Children's Hospital. One.

**Nurses.**

Birkdale Training Nurses' Home, Southport. One, Medical, Surgical, and Masseuse.
Bath Nurses' Home, 44, Rivers Street, Bath. One, Thoroughly Trained.
Jersey Institution for Hospital Trained Nurses. Several.
Victoria Home for Nurses, Bournemouth. Several.
Victoria Hospital, Burnley. Night Superintendent.—£30.
Kingston Nurses' Home, Baker Street, Hull. Several, Monthly, Surgical, and Massage.
Victoria Hospital Convalescent Home, Margate. One, Trained.
Workhouse Infirmary Association, 6, Adam Street, Adelphi. Several.

**Probationers.**

The Cottage Hospital, Llandudno. One.
Burton-on-Trent Infirmary. One.—£8.

---

THE number of chief operations performed at the Shropshire and North Wales Eye Hospital during last year was 210. Of 60 operations for cataract only two were unsuccessful.

---

THE Committee of the Hastings and St. Leonards Hospital must be very grateful to the dead benefactor whose legacy of £500 has enabled them to close their financial year with a surplus instead of a deficit.

---

AT the annual meeting of the Edinburgh Royal Maternity (Simpson Memorial) Hospital it was stated that the number of patients treated there during last year was 280, while 48 nurses were trained, and 176 students received clinical instruction.

---

PROFESSOR MATTHEW HAY has been elected Medical Officer of Health for Aberdeen. Several of the unsuccessful candidates purpose uniting in action to dispute the legality of the election, as by the terms of the advertisement of the vacant post the gentleman who received it was expected to give all his time to its duties, which Dr. Hay is not prepared to do.

---

THE Committee of Visitors of the new asylum for the West Riding of York, at Menston, near Leeds, lately advertised for a medical superintendent. Twenty-nine candidates offered themselves, and of these five or six were selected for further consideration. The choice ultimately fell on Dr. McDowall, Assistant Medical Officer at the South Yorkshire Asylum. The psychological prophets were right this time; and the appointment will give general satisfaction in the specialty.

MISS FORRESTER has been appointed to succeed Miss Lowe as Lady Superintendent of York County Hospital.

---

DURING last year the legacies received by St. Mary's Hospital, Manchester, amounted to £3,300, all of which, with the exception of £100 appropriated for income, has been invested.

---

THE annual general meeting of the Bengal Branch of the Countess of Dufferin's Fund, was held on February 6th, at the Bengal Town Hall. There was an influential gathering of ladies and gentlemen present, and the chair was occupied by his Honour the Lieutenant-Governor of Bengal. The hon. secretary, Mr. D. W. R. Edwards, said the report which had been printed and circulated had been adopted unanimously at a recent meeting of the Executive Committee. Among other things the report stated that great improvement had been effected in the Lady Dufferin Zenana Hospital by the opening of a ward for in-patients in July, and the conversion of the dispensary into a hospital. At the time of its opening the hospital contained only six beds, and two more had since been added. By the opening of these wards facilities for medical treatment have been afforded to a class of women who, either by their want of means or their strong prejudices, are debarred from all professional help, and the scope of the institution has thus been greatly widened.

---

THE third annual meeting of the National Association for Supplying Female Medical Aid to the Women of India was held recently, his Excellency the Viceroy in the chair. There was a large and brilliant gathering, including her Excellency the Countess of Dufferin, Lord and Lady Connemara, Sir Steuart and Lady Bayley, Sir Charles and Lady Aitchison, Sir Alexander and Lady Wilson, his Highness the Maharaja of Durbhunga, the Nawab Bahadur of Moorshedabad, the Maharaja of Vizianagram, the members of the Supreme and Local Councils, and others. The Hon. Mr. Scoble presented the report of the Central Committee, which he asked the meeting to accept and confirm, together with the accounts connected with it. He had to report success all along the line, and to say that the movement begun three years ago was now an established fact. Whereas last year the funds amounted to two lakhs of rupees, this year they amounted to six lakhs, of which four had been invested. A great deal of this amount was mainly attributable to the liberal response which was made to the Countess of Dufferin's appeal for Jubilee collections. The two objects which the National Association was designed to promote were medical tuition and medical relief. To these objects a third was now added, viz., that of supplying female nurses and midwives. Mr. Scoble bore testimony to the interest evinced in the movement by the Mercers' Company in London, whose good example he hoped many would be induced to emulate. With regard to medical relief he was glad to say that three native princes—the Nizam of Hyderabad, the Raja of Kuppurthula, and the Raja of Nanparah intended building hospitals as memorials of the Queen's Jubilee in their respective capitals. His Excellency the Viceroy, in a happy and appropriate speech closing the proceedings, said: "There is no one perhaps more capable than myself of bearing testimony to the constant and earnest attention which her Excellency is continually paying to your interests; for only too frequently, when the hard labours of my own office are concluded, and I repair to the retirement of my zenana for the purpose of seeking that repose which my conscience tells me I have earned, I am grievously disappointed by finding her Excellency so closely engaged upon the various matters connected with her 'fund,' tha she is unable to pay me any attention whatever."

